# Visual function restoration with a highly sensitive and fast Channelrhodopsin in blind mice

**DOI:** 10.1038/s41392-022-00935-x

**Published:** 2022-04-18

**Authors:** Fei Chen, Xiaodong Duan, Yao Yu, Shang Yang, Yuanyuan Chen, Christine E. Gee, Georg Nagel, Kang Zhang, Shiqiang Gao, Yin Shen

**Affiliations:** 1 Eye Center, Wuhan University Renmin Hospital, Wuhan, China; 2 Department of Neurophysiology, Institute of Physiology, Biocenter, University of Wuerzburg, Wuerzburg, Germany; 3 Institute for Synaptic Physiology, University Medical Center Hamburg Eppendorf, Hamburg, Germany; 4 Center for Biomedicine and Innovations, Faculty of Medicine, Macau University of Science and Technology and University Hospital, Macau, China; 5 Frontier Science Center for Immunology and Metabolism, and Medical Research Institute at School of Medicine, Wuhan University, Wuhan, China

**Keywords:** Translational research, Genetic engineering

**Dear Editor**,

Inherited and age-related retinal degenerative diseases cause progressive loss of photoreceptors, ultimately leading to blindness. Optogenetics is a promising strategy for restoring visual function through photosensitive proteins’ ectopic expression in surviving retinal neurons.^1^ Very recently, the optogenetic method with a red-shifted Channelrhodopsin was clinically applied for partial recovery of visual function in a blind patient.^2^ However, major obstacles to achieving optimal optogenetic vision restoration are either the low light sensitivity or the slow kinetics of existing rhodopsin-based optogenetic tools, which can be improved by molecular engineering to enhance the efficacy of fast Channelrhodopsins (ChRs). Here, we present a newly engineered ChR variant *Ps*CatCh2.0, engineered from *Ps*ChR,^3^ which displays inherently high Ca^2+^ and Na^+^ conductance and fast kinetics.^3,4^ We introduced a novel mutation *Ps*ChR L115C (*Ps*CatCh) to enhance its Ca^2+^ and Na^+^ permeability further and fused the cleavable N-terminal signal peptide Lucy-Rho (LR^5^ in Fig. 1a), in addition to a plasma membrane trafficking signal (T) and ER export signal (E), to improve its expression and plasma membrane targeting. *Ps*CatCh2.0 exhibited significant improvements in expression levels/plasma membrane targeting efficiency and a larger photocurrent (Fig. 1a, b, e). 100-fold less light intensity is needed to generate a similar photocurrent response with *Ps*CatCh2.0 than with CatCh (Fig. 1b), with BAPTA, Ca^2+^ currents of *Ps*CatCh2.0 were four times larger than those generated by CatCh (Fig. 1c, d), indicating that *Ps*CatCh2.0 is a highly effective excitatory tool for future clinical applications.

**Fig. 1:**
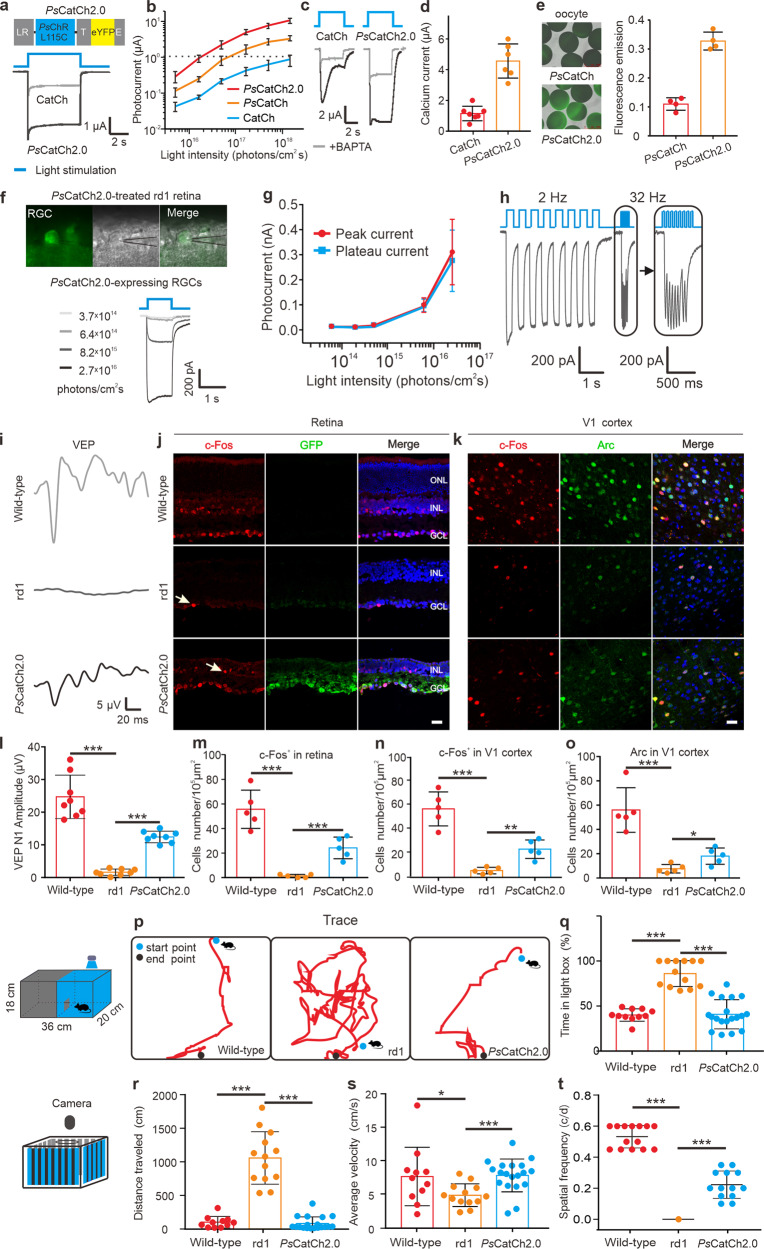
The characterizations of PsCatCh2.0, and the efficiency for vision restoration by optogenetics in rd1 mice. **a** Scheme of *Ps*CatCh2.0 and representative photocurrent traces of CatCh and *Ps*CatCh2.0 measured by two-electrode voltage clamp (TEVC) in Xenopus oocyte. **b** Stationary photocurrents of CatCh, *Ps*CatCh and *Ps*CatCh2.0 illuminated by different intensities of blue (450 nm) light. *n* = 3. **c** Representative photocurrent traces of CatCh and *Ps*CatCh2.0 in 80 mM CaCl_2_ pH 9.0 buffer with (both top traces) and without 10 mM BAPTA injection, holding at −100 mV. **d** Comparison of the CatCh and *Ps*CatCh2.0 calcium current. *n* = 6–7. **e** Fluorescence pictures (left) of *Ps*CatCh and *Ps*CatCh2.0-expressing oocytes and fluorescence emission values (right) of *Ps*CatCh and *Ps*CatCh2.0-expression oocytes. All measurements were done two days after injecting 20 ng cRNA into fresh oocytes. *n* = 4. **f** Representative image of whole-cell patch-clamp recording ganglion cell in *Ps*CatCh2.0-treated rd1 retinal slice, the light-evoked current traces of *Ps*CatCh2.0-expressed RGCs with 1 s light pulses at 470 nm under different light intensities measured as photons/cm^2^ s. **g** The light intensity and current response relationship were measured at peak and plateau currents. *n* = 5. **h** Temporal properties of *Ps*CatCh2.0 in retina induced photocurrents at increasing stimulation frequencies at a light intensity of 2.7 × 10^16^ photons/cm^2^ s of 470 nm blue light. **i** Representative VEP waveforms. **j** Representative co-labeling of c-Fos (red), an immediate early gene, with GFP (green) in vertical retinal sections. **k** Representative co-labeling of light-induced immediate early genes c-Fos (red) and Arc (green) in the V1 area of the visual cortex. **l** Graphed VEP N1 amplitudes. *n* = 8. Representative result of counting positive cells of c-Fos or Arc in the retina (**m**) and V1-visual cortex (**n, o**), respectively. All mice experiments under the light intensity of 4.7 × 10^14^ photons/cm^2^ s of 470 nm blue light. One-way ANOVA test, **P < 0.01, ***P < 0.001. Scale bars, 20 μm (**j**, **k**). *n* = 5. **p** Representative traces of the first time to find the hole in light/dark box. **q** Percentage of time spent in the light compartment under a light intensity of 4.7 × 10^14^ photons/cm^2^ s. *n* = 11–19. **r**, **d** Representative distance and average velocity of the first time to find the hole in light/dark box, one-way ANOVA test, ***P < 0.001). **t** The average spatial acuity of mice (t-test, ***P < 0.001). Stimulus light intensity 4.7 × 10^14^ photons/cm^2^ s. All data showed mean ± SD

The photosensitivity and kinetics of *Ps*CatCh2.0 were further investigated in vivo in rd1 mice. Notably, a low light intensity (3.7 × 10^14^ photons/cm^2^ s) evoked a 14.5 pA (14.5 ± 7.4, *n* = 5) current in *Ps*CatCh2.0-expressing RGCs in rd1 mice (Fig. 1g). It also presented a persistent periodic response that could follow up to 32 Hz light stimuli, without obvious desensitization (Fig. 1h), clearly outperformed MCO1 in kinetic aspect.^6^ Moreover, *Ps*CatCh2.0 could reliably induce action potentials firing at 100 Hz when expressing in the hippocampal neuron (Supplementary Fig. 1). We tested whether visual information input could be transmitted from the *Ps*CatCh2.0-treated retina to the brain in rd1 mice. We assessed the activity in the V1 cortex induced by light through c-Fos and Arc. Following 2 h of continuous light stimulation (470 nm, 4.7 × 10^14^ photons/cm^2^ s), both IEGs c-Fos (red) and Arc (green) were expressed in the light-stimulated retina and V1 cortex of wild-type and *Ps*CatCh2.0-expressing rd1 mice (Fig. 1j, k, m–o). In contrast, rd1 mice retina exhibited neither obvious light responses nor upregulation of IEGs in the visual cortex. Additionally, blue light flash visual evoked potential (VEP) recording in the visual cortex was performed. No obvious N1 amplitude in rd1 mice was recorded (1.6 ± 1.0 µV, *n* = 8) compared to the wild-type mice (−24.7 ± 6.7 µV, *n* = 8, Fig. 1i, l). In *Ps*CatCh2.0-treated rd1 mice, the N1 amplitude of VEP was restored to −12.4 µV (−12.4 ± 1.8 µV, *n* = 8), suggesting regained visual function after optogenetic treatment of blind mice.

Finally, we evaluated visually guided behavior in *Ps*CatCh2.0-treated rd1 mice. The fraction of time spent in light boxes, the distance and speed of movement for discovering the hole to the dark box were recorded. *Ps*CatCh2.0-treated rd1 mice in the blue-light chamber could easily find the hole entering to the dark box, with similar performance as the wild-type mice (percentage of time spent in the light box: *Ps*CatCh2.0, 40.8% ± 3.7 (*n* = 19); wild-type, 40.1% ± 2.1 (*n* = 11); rd1, 86.1% ± 4.0, (*n* = 13); one-way ANOVA; Fig. 1q). *Ps*CatCh2.0 also rescued the distance and average speed performance of rd1 mice to the wild-type level (Fig. 1r: distance (cm): wild-type, 101.9 ± 25.7, *n* = 11; *Ps*CatCh2.0, 82.3 ± 22.5, *n* = 19; rd1, 1058 ± 108.3, *n* = 13; Fig. 1s: average speed (cm/s): *Ps*CatCh2.0, 7.7 ± 0.6, *n* = 19; wild-type, 7.6 ± 1.3, *n* = 11; rd1, 4.8 ± 0.5, *n* = 13). Especially, *Ps*CatCh2.0-treated rd1 mice showed visual tracking behavior to the grating flash with an average peak spatial frequency of 0.22 ± 0.02 (c/d), compared to no response of the rd1 littermates, and 0.53 ± 0.02 c/d of the wild-type mice (Fig. 1t). Therefore, *Ps*CatCh2.0-treated rd1 mice improved visual acuity dramatically.

A light intensity of 4.7 × 10^14^ photons/cm^2^ s was all present to induce retinal, cortical and behavioral responses, which is safe for light therapy. In this study, *Ps*CatCh2.0 was expressed in retinal ganglion cells of blind rd1 mice. Visual acuity raised to 0.22 c/d, with a temporal resolution of at least 32 Hz. The faster and larger current *Ps*CatCh2.0 may be an optimal therapeutic option for the treatment of retinal degeneration. Furthermore, the blue-shifted action spectrum of *Ps*CatCh2.0 (Supplementary Fig. 2) provided the possibility to combine with red-shifted optogenetic tools^2^ to achieve colored vision restoration in the future.

## Supplementary information

Supplemental Material

## Data Availability

The data sets used for the current study are available from the corresponding author upon reasonable request.
