# Supplementary material for: Visual function restoration with a highly sensitive and fast Channelrhodopsin in blind mice
Source: Signal Transduct Target Ther. 2022 Apr 18;7:104. doi: 10.1038/s41392-022-00935-x (PMC9013715; doi:10.1038/s41392-022-00935-x)
Supplement: Supplementary file 1 — Supplemental Material [file 41392_2022_935_MOESM1_ESM.docx]

**Supplemental Material and Figures for**

**Visual function restoration with a highly sensitive and fast Channelrhodopsin in blind mice**

Fei Chen^1^#, Xiaodong Duan^2^#, Yao Yu^1^#, Shang Yang^2^, Yuanyuan Chen^1^, Christine E Gee, Georg Nagel^2^, Kang Zhang^4^*, Shiqiang Gao^2^*, Yin Shen^1.5^*

^1^Eye Center, Wuhan University Renmin Hospital, Wuhan, China.^2^Department of Neurophysiology, Institute of Physiology, Biocenter, University of Wuerzburg, Wuerzburg, Germany.^3^Institute for Synaptic Physiology, University Medical Center Hamburg Eppendorf, Hamburg, Germany. ^4^Center for Biomedicine and Innovations, Faculty of Medicine, Macau University of Science and Technology and University Hospital, Macau, China. ^5^Medical Research Institute, Wuhan Unveristy, China.

# These authors contributed equally to this work

*Correspondence: Yin Shen ([yinshen@whu.edu.cn](mailto:yinshen@whu.edu.cn)), Shiqiang Gao ([gao.shiqiang@uni-wuerzburg.de](mailto:gao.shiqiang@uni-wuerzburg.de)) or Kang Zhang ([Kang.zhang@gmail.com](mailto:Kang.zhang@gmail.com)).

**This PDF file includes:**

Materials and Methods

Figures. S1 to S2

**Materials and methods**

**Plasmids and RNA Generation for *Xenopus laevis* Oocyte Expression**

*Ps*ChR (from *Platymonas subcordiformis*, Accession No.: JX983143) was synthesized by GeneArt Strings DNA Fragments (LifeTechnologies, Thermo Fisher Scientific), according to the published amino acid sequences, with the codon usage optimized to *Mus musculus*. CatCh was obtained in our lab. The synthesized DNA segment was inserted into the pGEMHE vector with N-terminal BamHI and C-terminal XhoI restriction sites. Yellow fluorescent protein (YFP), together with a plasma membrane trafficking signal (KSRITSEGEYIPLDQIDINV) beforehand and an ER export signal (FCYENEV). Afterward, the YFP was attached to the C-terminal end. Mutations were made by QuikChange Site-Directed Mutagenesis. The sequence was confirmed by DNA sequencing. Plasmids were linearized by NheI digestion and used for in vitro generation of cRNA with the AmpliCap-MaxT7 High Yield Message Maker Kit (Epicentre Biotechnologies).

**Two-Electrode Voltage-Clamp Recordings of *Xenopus laevis* Oocytes**

cRNA-injected oocytes were incubated in ND96 solution (in mM) (96 NaCl, 5 KCl, 1 MgCl_2_, 1 CaCl_2_, 5 HEPES, pH 7.4) containing 1 µM *all-trans*-retinal at 16^◦^C. Two-electrode voltage-clamp (TEVC) recordings were performed with solutions, as indicated in figures, at room temperature. For experiments with external Ca^2+^, we blocked activation of the Ca^2+^-activated endogenous chloride channels of oocytes by (BAPTA) injection. We injected 50 nl 200 mM of the fast Ca^2+^ chelator BAPTA (potassium-salt) into each oocyte (~10 mM final concentration in the oocyte), incubated for 90 mins at 16^◦^C and then performed the TEVC measurement at room temperature. Twenty nanograms of cRNA were injected into *Xenopus* oocyte for all the constructs. Photocurrents were measured two days after injection. For Fig. 1 and Fig. S1, measurements were performed in standard ORI solution with BaCl_2_ instead of CaCl_2_ (in mM) (110 NaCl, 5 KCl, 2 BaCl_2_, 1 MgCl_2_, 5 HEPES and pH 7.6).

**Protein Quantification by Fluorescence and Fluorescence Imaging**

All expression levels of channelrhodopsin variants in oocytes were quantified by the fluorescence emission values of the YFP-tagged protein. Fluorescence emission was measured at 538 nm by a Fluoroskan Ascent microplate fluorometer (Thermo Scientific) with 485 nm excitation. Fluorescence pictures of *Xenopus* oocytes were taken under 5 x objective with a Leica DM6000 confocal microscope after two days’ expression. Oocytes were put in a 35 x 10 mm petri dish (Greiner GBO) containing ND96 for imaging. Excitation was done using 496 nm laser light. Fluorescence emission was detected from 520 nm to 585 nm.

**Animals and Viral Vectors**

The C57BL/6J was purchased from Vital River Laboratory Animal Technology Co., Ltd. (Beijing, China). The rd1 mice was given by Ying Xu, who from Guangdong-Hongkong-Macau Institute of CNS Regeneration, Ministry of Education CNS Regeneration Collaborative Joint Laboratory, Jinan University, China. Wistar rats (Envigo) were bred at the University Medical Center (UKE), Hamburg. All procedures were approved by the Behörde für Justiz und Verbraucherschutz (BJV)-Lebensmittelsicherheit und Veterinärwesen, Hamburg and the animal care committee of the UKE.PsCatCh2.0 were cloned into the recombinant AAV (rAAV) vector cassette and fused in the frame to EGFP (*Ps*CatCh2.0-EGFP). Expression was driven by a cytomegalovirus (CMV) promoter. Viral vectors were packaged into AAV2/2 serotype for PsCatCh2.0, and affinity purified by GENE (Genechem Co., Ltd. Shanghai, China), and rAAV-CAG-hChR2 (H134R)-mCherry-WPRE-hGH polyA (5.13×1012 v.g/ml, 2/9 serotype) was purchased from BrainVTA (Wuhan, China).

**Patch Clamp Recording in Hippocampal Neurons**

Single CA3 neurons in rat organotypic hippocampal slice cultures were electroporated with 10 ng/µl pAAV-syn-*Ps*CatCh and 10 ng/µl pAAV-syn-mKate2^1,2^. After 4-15 days of expression whole cell recordings were made from the neurons identified by the mKate2 fluorescence as the *Ps*CatCh label was not visible. The extracellular solution was (in mM): NaCl 119, NaHCO3 26.2, D-glucose 11, KCl 2.5, NaH2PO4 1, MgCl2 4, CaCl2 4, pH 7.4, 310 mOsm kg-1, saturated with 95% O_2_/ 5% CO_2_. Picrotoxin (100 µM), NBQX (10 µM) and CPPene (1 µM) were added to block synaptic responses. The intracellular solution contained (in mM): K-gluconate 135, HEPES 10, EGTA 0.2, Na2-ATP 4, Na-GTP 0.4, MgCl2 4, ascorbate 3, Na2- phosphocreatine 10, pH 7.2, 295 mOsm kg-1. Series resistance was 7-19 MΩ and recordings were discontinued if it changed more than 30%. The bridge was balanced during current clamp recordings. Voltage clamp was to -70 mV. The liquid junction potential was compensated (-14.4 mV). Photocurrents were analysed in Matlab.

**Viral Vector Injection**

All animal experiments and procedures were approved by Institution Animal Care and Use Committee of Wuhan University, and they were performed in accordance with the NIH Guide for the Care and Use of Laboratory Animals. Intravitreal injections of viral vectors were administered to 4-week-old wild-type C57BL/6J and rd1 mice. Briefly, the animal was anesthetized with an intraperitoneal injection of a mixture of 100 mg/Kg Ketamine and 12 mg/Kg xylazine. Viral vectors (rAAV2/2-CMV-*Ps*CatCh2.0-EYFP, 1.5 µl) at titer of 2.1 ×10^12^ vg/ml or vehicle (saline) were intravitreally injected into both eyes of each animal. In order to improve the transfection efficiency of the virus, we had another the same injection 2 weeks later. The virus vectors were injected with a microinjection of Nanoject III programmable Nanoliter Injector (Drummond Scientific, USA) using glass micropipettes (WPI, USA). All experiments were performed at least 1 month after the last virus injection. Animals were euthanized by CO_2_ asphyxiation followed by decapitation for electrophysiological recording and immunostaining.

**Immunostaining and Quantitative Cell Density Measurements**

Enucleated eyes were fixed with 4% paraformaldehyde at room temperature for 30 min. Fluorescence expression was examined in flat-mount retinas and retinal vertical sections. The expression of *Ps*CatCh2.0 in retinal was examined by co-labeling with chicken anti-GFP (1:1,000, abcam, USA), rabbit anti-Brn3a (1:800, Synaptic System, Germany), rabbit anti-RBPMS (1:500, Phosphosolutions, USA), rabbit-anti-Calbindin (1:5000, SWANT, Switzerland), rabbit anti-Ap2α (1:500, abcam, UK), sheep anti-Chx10 (1:500, Exalpha, USA). The secondary antibodies were conjugated with Alexa Fluor 594 (1:500, Jackson, USA), Alexa Fluor 488 (1:500, Jackson, USA). Cell nuclei were revealed with 4’, 6-diamidino-2-phenylindole (DAPI, 1:100, Life Technologies). The densities of cells and *Ps*CatCh2.0 expressing cells were measured by assessing specific marker-labeled and dual GFP/specific marker-labeled cells, respectively. All the retinal fluorescence images were captured using the confocal microscopy (Olympus FV1200). The cell numbers were manually counted with Adobe Photoshop CS5.

Before Immunostaining IEGs in Retinal, superior colliculus and V1 area of the visual cortex, mice were dark-adapted for 12 hours before experiment, then the mice were placed in the open top cages and freely moved for 2 hours under the blue light with a light intensity of 4.7 × 10^14^ photons/cm^2^s on the bottom of the box. After the end of illumination, the animal was anesthetized with an intraperitoneal injection of a mixture of 100 mg/Kg Ketamine and 12 mg/Kg xylazine. When the anesthesia was sufficient, we began to do cardiac perfusion in the mice. First, with PBS solution for 15 minutes to fully wash the blood of the tissues, and then with the 4% paraformaldehyde for 10 minutes. The eyeballs of the mice were removed and fixed in 4% paraformaldehyde for 20 minutes at room temperature, while the brain was placed in 4% paraformaldehyde and fixed in 4^◦^C refrigerator overnight. The retina was dehydrated in 30% sucrose in refrigerator at 4^◦^C for 12 hours, while the brain was dehydrated in 30% sucrose for 3 days. The retina was cut into 14 μm and the brain was cut into 50 nm with a freezing microtome (Leica CM 1950, Germany) for immunofluorescence staining. The retina was blocked with 4% BSAT for 1 hour at room temperature, and the brain was blocked in a 4^◦^C refrigerator for 12 hours. The primary antibody was incubated in a 4^◦^C refrigerator for 2 days. The secondary antibody was incubated for 2 hours at room temperature. DAPI was incubated at room temperature for 5 minutes. Chicken anti-GFP (1:1,000 for retina; Abcam, USA), rabbit anti-c-Fos (1:100 for retina; 1:500 for brain; Cell Signaling Technology, USA), mouse anti-Arc (1:250 for brain; Santa Cruz Biotechnology, USA). The secondary antibodies were conjugated to Alexa 594 and Alexa 488 (1:500, Jackson, USA).

**Patch clamp recordings for the retinal slice of *Ps*CatCh2.0 treated rd1 mice**

*Ps*CatCh2.0 treated rd1 mice were euthanized by CO_2_ asphyxiation followed by decapitation for electrophysiological recording. The retina was quickly dissected out of the extracellular fluid pre-filled with 95% O_2_ + 5% CO_2_ for at least 30 minutes. Later, we carefully cleaned the vitreous, then attached the RGC layer to the Millipore filter paper and quickly cut the retina into a 150 nm thickness on a manual slicer (Stoelting Tissue Slicer, USA). Recording in the whole-cell configuration were made using standard procedures at room temperature about 25^◦^C. The extracellular recording solution contained the following (in mM): 125 NaCl, 2.5 KCl, 1 MgSO_4_, 2 CaCl_2_, 1.25 NaH_2_PO_4_, 26 NaHCO_3_, 20 glucose, pre-fill mixture gas of 95% O_2_ + 5% CO_2_ for at least 30 minutes before use. The electrode solution contained the following (in mM): 115 CsCH_3_O_3_, 20 CsCl, 2.5 MgCl_2_, 0.6 EGTA, 10 HEPES, 4 ATP-Mg, 0.4 GTP-Na, 10 phosphocreatine, with the PH adjusted to 7.2 using CsOH. The light stimulation system and the patch clamp device were the same as the HEK 293T cell experiment. But, patch electrodes were made from borosilicate glass pulled to 5-7 MΩ.

**Flash Visual evoked potential Recording**

Flash visual evoked potential (FVEP) was used to assess visual function at 1.5 month after virus intravitreal injection from the mice dura using FLASH STIMULATOR (IRC, Chongqing, China). Electrodes were implanted as explained earlier. Briefly, mice were anesthetized with an intraperitoneal injection of a mixture of 100 mg/Kg Ketamine and 12 mg/Kg xylazine. The mice head was shaved and the dorsal surface of the skull exposed. And then, the mice head was fixed with a brain stereotaxic apparatus (RWD, Shenzhen, China). The 0.25 mm diameter Ag wire electrode was implanted on the right primary visual cortex (recording electrode, 3.6 mm caudal to bregma, 2.3 mm lateral to lambda) 48 h before flash VEP experiment. Then, mice were dark-adapted overnight, dilated with compound tropicamide eye drops (0.5% topicamide and 0.5% deoxyepinephrine hydrochloride) for 5 min, and anesthetized with an intraperitoneal injection of a mixture of 100 mg/Kg Ketamine and 12 mg/Kg xylazine. A reference electrode was inserted under the skin between the eyes whereas the ground electrode was clamped to the tail. Flash light stimulation was delivered by FLASH STIMULATOR (IRC, Chongqing, China), for 64 times repetitive light stimulus (2800 μs, blue light, 5.0 cds/m^2^). Recording was done with 2000 Hz sampling rate, bandpass filtering between 3.0 and 70.0 Hz. RetiMINER 4.0 software (IRC, Chongqing, China) was used to generate and record the readings and obtain the data table for N1 amplitude.

**Light-induced behavior of *Ps*CatCh2.0-treated rd1 mice in light/dark box and optomotor assays**

The light/dark box is composed of two boxes of the same dimensions (18cm (l) × 20 cm (b) × 18 cm (h)), with a hole (7 cm (b) × 5 cm (h)) in the middle. The light compartment was fitted with 470 nm LED source (Mightex, Canada), all mice were age-matched, and their ages ranged 10-12 weeks at time of testing. Wild-type, *Ps*CatCh2.0 treated rd1 mice, and rd1 mice were subjected to 5 minutes trials during the light/dark box at light intensity 4.7 × 10^14^ photons/cm^2^s of blue (470 nm) light. The mice were dark adaptation for 2 hours before the experiment. All behavioral experiments were conducted between 18:00 and 21:00. When the experiment began, the mice were left alone in light box and allowed to explore freely. The position of the mice head was used to analyze the movement in light/dark box. The movies were analyzed using animal tracking system software Ethovision XT (Noldus Information Technology, VA). Data were collected on the track, distance and average speed of the mice when they first found the hole in the light box and entered the dark box. These data were then analyzed and plotted in Prism 7 (GraphPad Software, La Jolla, CA). The significance was assessed using a one-way ANOVA, and an α-level of P < 0.05 was considered to be significant. Next, OMRs were examined using a homemade optomotor system. Four computer monitors (Lenovo, L1900pA) faced a 17.5 cm high platform above a mirrored floor under a likewise mirrored lid. A positive OMR, or head tracking, was determined by repeated head angular movements in the directing of the rotating drum. Head tracking was tested for both clockwise and counterclockwise directions. The spatial frequency of the test grating was changed in steps until the highest spatial tracking frequency was identified as the threshold (visual acuity). The experimental data were confirmed by a second experimenter.

**Figures**

**
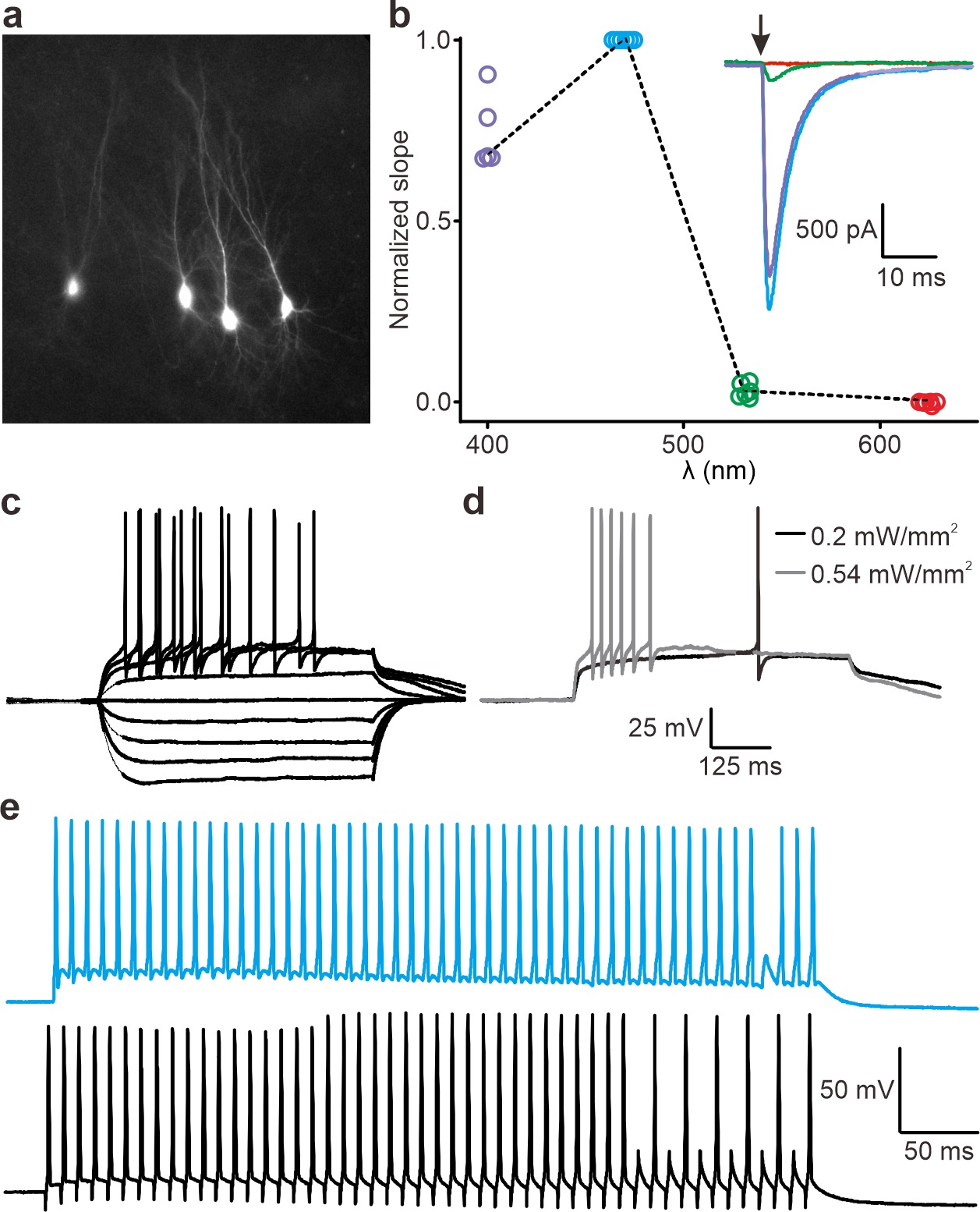
**

**Figure S1. *Ps*CatCh2.0 enables high frequency optical firing of hippocampal neurons.**

(a) Fluorescence image of hippocampal CA3 neurons in an organotypic slice cultures co-expressing *Ps*CatCh2.0 and mKate2. Shown is mKate2 fluorescence. Scale bar 50 µM. (b) Wavelength dependence of photocurrents evoked by 2 ms 2 mW/mm^2^ light flashes of violet (400 nm), blue (470 nm), green (530 nm) and red (625 nm) light from 6 neurons voltage clamped at -70 mV. The dotted line connects the medians. Inset shows photocurrents recorded from one neuron. Arrow indicates time of light flash. (c) Whole cell membrane responses to -400 pA to 400 pA current injection steps. Step size 100 pA, 600 ms. Scale bars in (d) apply. (d) Response of the same neuron as in (c) to 600 ms 470 nm light pulses. (e) Upper trace shows action potentials induced by 50 light flashes at 100 Hz in a different neuron. Each flash is 2 ms, 10 mW/mm^2^, 470 nm. Lower trace action potentials evoked in the same neuron by 2 ms 4500 pA current injections (50 at 100 Hz). Note that only one light flash failed to evoke an action potential whereas the neuron failed to fire 6 times during current injection steps.


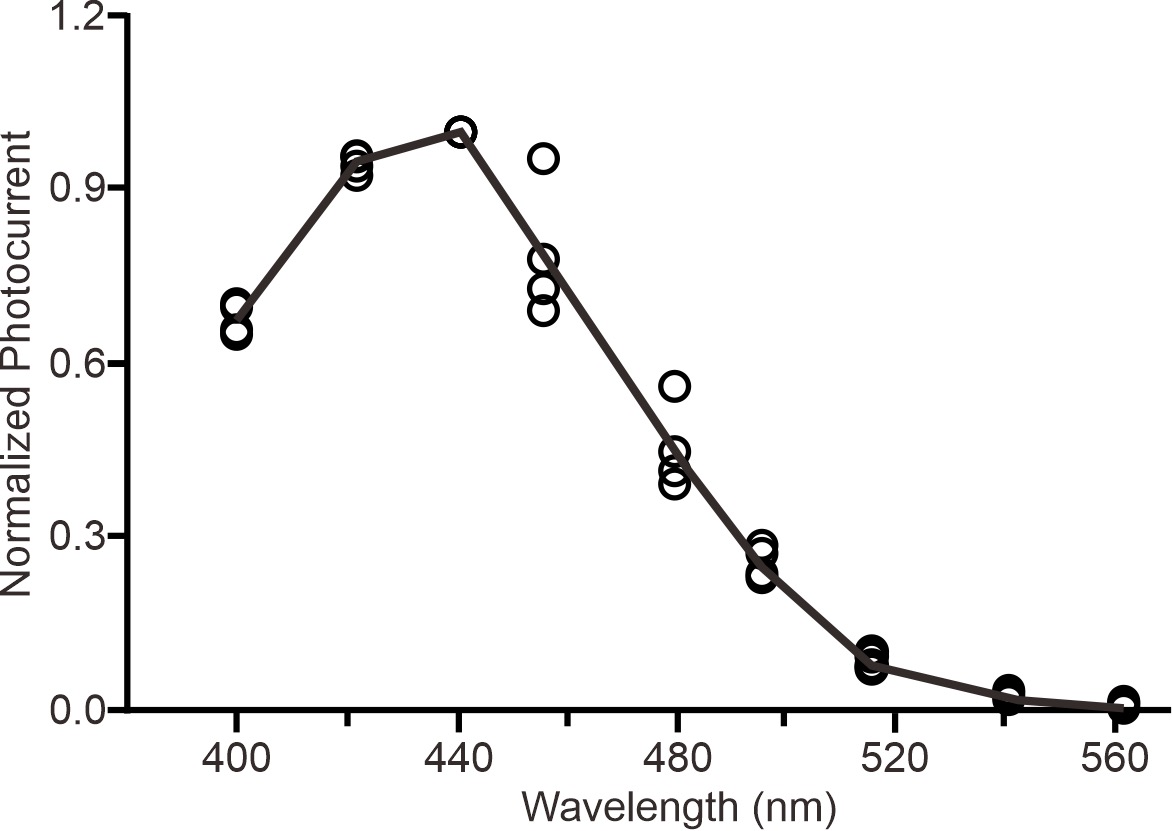


**Fig S2. The blue-shifted action spectrum of *Ps*CatCh2.0**

For action spectra of *Ps*CatCh2.0 (n = 4), light of different wavelengths was obtained by narrow bandwidth interference filters (Edmund Optics) together with a PhotoFluor II light source (89 North). The action spectrum of *Ps*CatCh2.0 was normalized to photostimulation at 441 nm.

**reference**

1 Wiegert, J. S., Mahn, M., Prigge, M., Printz, Y. & Yizhar, O. Silencing Neurons: Tools, Applications, and Experimental Constraints. *Neuron* **95**, 504-529, doi:10.1016/j.neuron.2017.06.050 (2017).

2 Gee, C. E., Ohmert, I., Wiegert, J. S. & Oertner, T. G. Preparation of Slice Cultures from Rodent Hippocampus. *Cold Spring Harb Protoc* **2017**, doi:10.1101/pdb.prot094888 (2017).
